# Post-Operative Atrial Fibrillation: Novel Predictive Value of CT-Derived Adipose Tissue Density in Minimally Invasive Mitral Surgery

**DOI:** 10.33549/physiolres.935754

**Published:** 2025-12-01

**Authors:** Matej PEKAR, David VICIAN, Otakar JIRAVSKY, Bogna JIRAVSKA GODULA, Piotr BRANNY, Radim SPACEK, Marek KANTOR, Monika SKOTAKOVA, Jan CHOVANCIK, Libor SKNOURIL, Jan NOVAK

**Affiliations:** 1 Complex Cardiovascular Center, Hospital AGEL Trinec-Podlesi, Trinec, Czech Republic; 2 Department of Physiology, Faculty of Medicine, Masaryk University, Brno, Czech Republic; 3 Department of Cardiac Surgery, Faculty of Medicine, Palacky University, Olomouc, Czech Republic; 4 Faculty of Medicine Palacky University, Olomouc, Czech Republic; 5 Third Faculty of Medicine Charles University, Praha, Czech Republic; 6 Biostatistics Department, St. Anne’s University Hospital in Brno, Brno, Czech Republic; 7 Faculty of Medicine, University of Ostrava, Ostrava, Czech Republic; 8 Faculty of Medicine, Masaryk University, Brno, Czech Republic; 9 Second Department of Internal Medicine, St. Anne’s University Hospital in Brno, Brno, Czech Republic

**Keywords:** Postoperative atrial fibrillation, Body composition, Computed tomography, Mitral valve surgery, Metabolic risk factors

## Abstract

Postoperative atrial fibrillation (POAF) remains a significant complication following minimally invasive thoracoscopic mitral valve surgery (MITMVS), yet current risk prediction models inadequately capture the underlying metabolic determinants of arrhythmogenesis. We investigated whether computed tomography (CT)-derived body composition parameters, as markers of metabolic status, could predict POAF risk in patients undergoing mitral valve repair. We retrospectively studied 104 consecutive MITMVS patients (2014–2023). Preoperative CT scans quantified skeletal muscle index, muscle density, and visceral/subcutaneous adipose tissue. Patients were grouped by preexisting atrial fibrillation (AF) with concurrent Maze (n=48) vs. no AF history (n=56). The primary outcome was POAF development. Higher visceral (VAT) and subcutaneous (SAT) adipose tissue density showed associations with increased POAF odds in multivariable analysis (VAT: OR 1.075, 95 % CI: 1.010;1.149, p=0.026; SAT: OR 1.073, 95 % CI: 1.011;1.146, p=0.025). Quartile analysis revealed a striking 5.5-fold increased POAF risk in the highest VAT density quartile compared to the lowest (42.3 % vs. 7.7 %). Notably, the relationship between intramuscular adipose tissue (IMAT) density and POAF differed between groups (interaction p=0.029), with a positive association in patients without prior AF (OR 1.167, 95 % CI: 1.011;1.377, p=0.047), but no significant relationship in those with preexisting AF (p=0.175). CT-derived tissue quality parameters, particularly VAT density, demonstrate robust associations with POAF risk following MITMVS. These findings establish preoperative CT-based metabolic assessment as a promising tool for perioperative risk stratification without additional testing burden.

## Introduction

Cardiometabolic disorders, including metabolic syndrome and its features (inflammation, insulin resistance, dyslipidemia, hypertension), are increasingly linked to atrial fibrillation (AF). These conditions promote AF through adverse atrial remodeling, abnormal adipose deposition, inflammatory cytokine production, oxidative stress, and neurohumoral activation [1].

Postoperative atrial fibrillation (POAF) is the most common perioperative arrhythmia, affecting up to 50 % of cardiac surgery patients, yet metabolic factors remain underrepresented in current risk models [2]. Recent work shows that adipose tissue distribution and activity – *via* myocardial infiltration, paracrine effects, and systemic signaling – can drive arrhythmogenesis [3,4]. Notably, 20–50 % of cardiac surgery patients develop POAF, with 40 % experiencing multiple episodes peaking around postoperative days 2–3 [5,6].

Inflammation and metabolic dysregulation coincide with the peak incidence of POAF [6). Additional contributors include atrial refractoriness, oxidative damage, and re-entry circuits [7]. Although prophylaxis (e.g., beta-blockers or amiodarone) and minimally invasive surgery have shown promise [8], refining patient selection and risk stratification remains challenging.

Given metabolic dysfunction’s central role in POAF, computed tomography (CT)-derived skeletal and adipose tissue parameters offer crucial insights [9,10]. The L3 vertebral level reliably reflects whole-body composition, enabling both quantitative and qualitative analysis *via* radiodensity ([11,12]. Beyond mere energy storage, adipose tissue acts as an active endocrine organ, shaping inflammation and systemic function [13,14].

The third lumbar vertebral (L3) CT parameters reflect body composition and metabolic health [15,16]. Elevated visceral, subcutaneous, and intramuscular fat densities (“metabolically unfavorable”) correlate with inflammation, fibrosis, and insulin resistance [17,18], mirroring AF’s structural and electrical remodeling [19,20]. Insulin resistance drives ectopic fat accumulation, further impairing metabolic function. Low skeletal muscle index (SMI) indicates sarcopenia, while high intramuscular fat suggests poor muscle quality [21].

Our prior work showed these CT metrics predict mortality risk: higher adipose densities correlate with cardiovascular and cancer mortality, while lower muscle mass reduces overall survival, especially in men undergoing transcatheter aortic valve implantation (TAVI) [15,16]. Body composition also significantly affects outcomes in colorectal [22], breast [23], ventral hernia [24], bariatric [25], and cardiac [26] surgeries.

Mitral valve surgery involves both direct cardiac manipulation and systemic metabolic stress [27], making it a compelling setting to explore these factors. Concurrent Maze procedures for patients with AF add complexity, potentially modifying metabolic-arrhythmia relationships [28]. While minimally invasive approaches offer advantages over sternotomy, precise patient selection and risk stratification remain pressing challenges [2,27].

In this study, we use routine preoperative CT to assess how tissue-specific metabolic parameters – especially visceral adipose tissue (VAT) and subcutaneous adipose tissue (SAT) density – relate to POAF after mitral valve surgery. This approach builds on evidence linking metabolic dysfunction and arrhythmogenesis, offering practical insights for perioperative risk stratification using existing clinical imaging.

## Methods

### Study population and design

This single-center retrospective cohort study was registered on ClinicalTrials (NCT06707506) and approved by the institutional ethics committee (EK 65/24) in adherence to the Declaration of Helsinki principles. We reviewed records of consecutive patients who underwent minimally invasive thoracoscopic mitral valve surgery (MITMVS) between January 2014 and November 2023 at the Complex Cardiovascular Center, Hospital AGEL Trinec-Podlesi.

Inclusion criteria comprised: 1. age ≥18 years, 2. preoperative CT scan with coverage of L3 vertebra level performed within 30 days before surgery, 3. isolated MITMVS or MITMVS with concomitant Maze procedure, and 4. complete clinical and follow-up data availability. Exclusion criteria included: 1. emergency procedures, 2. concomitant cardiac procedures other than Maze, 3. conversion to sternotomy, 4. prior cardiac surgery, and 5. poor CT image quality precluding accurate tissue analysis.

From 114 consecutive eligible patients, the final study cohort comprised 104 patients after excluding ten patients with permanent preexisting AF who did not undergo Maze procedure (Group C). These patients were excluded due to their distinct pathophysiological profile of permanent AF (continuous AF before surgery without Maze treatment and expected continuous AF postoperatively) and limited sample size for meaningful statistical analysis. The remaining patients were stratified into two groups based on their preoperative AF status and treatment approach: those without previous AF history (Group A, n=56) and those with preexisting AF who underwent concurrent Maze procedure (Group B, n=48) (patient selection and group assignment is visually described in Suppl. Fig. 1). This stratification enabled investigation of body composition parameters in both AF-naïve patients and those receiving comprehensive AF treatment through Maze procedure.

### CT acquisition and body composition analysis

**Fig. 1:**
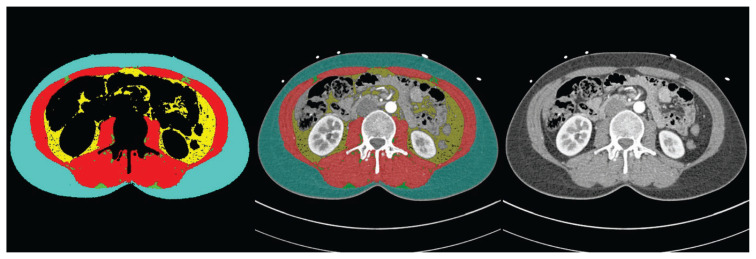
CT-Based Body Composition Analysis at L3 level in Mitral Valve Surgery Patients: Automated Segmentation and Original CT Images. Representative images from a single patient demonstrating automated body composition analysis at the third lumbar vertebra (L3) level. Left: Segmented image showing subcutaneous adipose tissue (turquoise), visceral adipose tissue (yellow), skeletal muscle (red), and intramuscular adipose tissue (green). Middle: Overlay of segmentation map on the original CT scan showing anatomical correlation of tissue compartments. Right: Original axial CT scan at L3 level used for automated tissue segmentation. This automated analysis enables objective quantification of body composition parameters including tissue areas and radiodensity measurements in Hounsfield units.

Preoperative CT imaging employed Siemens Somatom scanners (Definition pre-2016, Drive post-2016) with standardized acquisition protocols, including ECG gating and tri-iodinated non-ionic contrast medium administration. The analysis focused on L3 level using automated segmentation through deep learning algorithms AutoMATiCA [11], which is a UNET network for biomedical image segmentation, and achieved high accuracy across 893 patients (80 % training, 10 % validation, 10 % testing). Test results showed excellent Dice Similarity Coefficient scores comparing human vs. network segmentations, with ~350 ms processing time per scan. The system quantified cross-sectional areas (in cm^2^) and radiodensity (Hounsfield Units, HU) of skeletal muscle, intramuscular adipose tissue (IMAT), VAT and SAT. All tissue areas were normalized to height squared (cm^2^/m^2^) to generate standardized indices (Fig. 1).

All CT scans were acquired as part of routine preoperative surgical planning according to standardized institutional protocols, typically performed within the weeks preceding scheduled surgery. No metabolic, dietary, or weight-loss interventions were implemented between CT acquisition and surgical procedure. As CT-derived tissue radiodensity measurements reflect chronic metabolic status and tissue composition that evolves over months rather than acute changes occurring over days to weeks, the timing variability inherent in routine preoperative imaging is unlikely to have meaningfully affected body composition parameters.

### Metabolic parameter definition and analysis

Tissue quality parameters were defined based on radiodensity measurements, with specific attention to adipose tissue compartments as indicators of metabolic status. IMAT density served as a marker of muscle quality and local metabolic activity, while VAT and SAT density provided insights into systemic metabolic status. The relationship between these parameters and traditional metabolic markers (such as body mass index, BMI) was assessed to validate their role as metabolic indicators.

### Surgical approach

All procedures were performed under general anesthesia using standardized MITMVS protocols. Peripheral cardiopulmonary bypass was established *via* common femoral vessels. The procedure was conducted through a right mini thoracotomy (4–6 cm incision) in the 4^th^ intercostal space with a separate camera port placed one to two intercostal spaces above. After pericardiectomy and placement of an aortic root cardioplegia catheter, myocardial protection was achieved using antegrade cold blood cardioplegia following aortic cross-clamping.

Access to the left atrium was obtained through a standardized approach *via* the interatrial groove (Waterston’s groove). The left atrial incision extended from the area superior to the right superior pulmonary vein downward to the right inferior pulmonary vein. In cases requiring concurrent Maze procedure, this access incision was integrated into the ablation set to optimize procedural efficiency while maintaining consistent anatomical landmarks. All ablation procedures followed a standardized protocol using a CryoICE® probe (AtriCure, Inc.) at −60 °C with consistent application times (120 s per lesion).

For patients undergoing concurrent Maze procedure, a complete left atrial lesion set was performed, consisting of: 1. pulmonary vein box isolation, encompassing all pulmonary vein ostia and the majority of the posterior left atrial wall with continuous encircling lesions, 2. connecting lesions from the box to the mitral annulus in the left inferior pulmonary vein region (left atrial isthmus line), and 3. connecting lesion from the left superior pulmonary vein to the left atrial appendage. The left atrial appendage was routinely occluded with double-layer running 4–0 polypropylene suture.

### Clinical outcomes assessment

The primary outcome was POAF, defined as new-onset AF lasting more than 48 h or requiring medical intervention during the index hospitalization. POAF was assessed through continuous telemetry monitoring during the first 72 h postoperatively, followed by regular 12-lead ECG recordings until discharge. Secondary outcomes included perioperative complications (excessive bleeding requiring transfusion, prolonged ventilation >24 h), intensive care unit length of stay, and all-cause mortality. Clinical follow-up was conducted through August 1, 2024, using a combination of in-person visits and electronic health record review.

### Statistical analysis

Statistical analysis was performed using R programming language in R Studio integrated development environment. Results with p-values less than 0.05 were considered statistically significant. Continuous variables are presented as mean ± standard deviation (SD) or median (interquartile range, IQR). Categorical variables are presented as counts with percentages. Normality of continuous variables was assessed using the Shapiro-Wilk test and graphically assessed using Q-Q plots and histograms.

Prior to conducting the main analyses, we assessed for potential interactions between body composition parameters and patient groups to determine the appropriate analytical strategy. Where significant interactions were identified, indicating that the relationship between a tissue parameter and outcomes differed between groups, separate subgroup analyses were performed. For parameters showing no significant interactions, analyses were conducted on the combined cohort with appropriate adjustments for demographic factors. This approach ensured that the relationships between tissue parameters and clinical outcomes were analyzed in a manner that accurately reflected their underlying patterns within the patient population.

For statistical comparisons, Spearman’s correlation coefficient was used to evaluate correlations between variables. Linear regression and logistic regression were used to identify variables associated with outcomes. Chi-Square test was used to assess relationships between categorical variables. Student’s *t*-test or Mann-Whitney test was used to assess the difference in continuous variables between groups. Cox proportional hazards model was used to identify variables associated with overall survival. Odds ratios (OR), hazard ratios (HR), and 95 % confidence intervals (CI) are reported for regression analyses. Reverse Kaplan-Meier was used to calculate median follow-up time.

For the primary outcome of POAF, interaction terms between body composition parameters and preoperative AF status (Group A vs. B) were tested. Parameters showing no significant interaction (p>0.05) were analyzed in the combined cohort with adjustment for age and sex. Parameters with significant interaction were analyzed separately within each group, as the relationship with POAF differed by preoperative AF status.

For secondary outcomes (intubation time, intensive care unit (ICU) length of stay, blood loss), both univariable and multivariable linear regression models adjusting for age and sex were performed. Given the exploratory nature of these analyses, no formal correction for multiple comparisons was applied.

Additional multivariable models were constructed to assess potential confounding by clinical comorbidities (hypertension, diabetes mellitus, respiratory disease) and baseline cardiac function parameters. These covariates did not materially alter the associations between CT-derived parameters and outcomes, and were not retained in final models to avoid overfitting given the sample size.

## Results

### Patient characteristics and study population

Our analysis encompassed 104 consecutive patients undergoing MITMVS between 2014–2023. The cohort demonstrated a mean age of 62.99±11.26 years with male predominance (56.7 %, n=59). Among cardio-vascular risk factors, hypertension was most prevalent (79.8 %, n=83), followed by respiratory comorbidities (6.7 %, n=7) and diabetes mellitus (4.8 %, n=5). Preexisting AF was documented in 46.2 % (n=48) of cases (Table S1).

### CT-derived body composition analysis

Body composition analysis revealed distinct tissue-specific patterns. Skeletal muscle assessment showed a mean SMI of 48.26±8.97 cm^2^/m^2^ with average density of 36.81±7.32 HU. Adipose tissue compartments demonstrated characteristic distributions with standardized areas (VAT index: 50.97±26.38 cm^2^/m^2^, SAT index: 64.07±28.42 cm^2^/m^2^) and density measurements reflecting metabolic status (VAT: −100.63±7.11 HU, SAT: −106.86±7.62 HU). Comparative analysis of baseline characteristics and metabolic parameters between patients without prior AF (Group A) and those with preexisting AF undergoing concurrent Maze procedure (Group B) is summarized in Table 1.

### Primary outcome analysis: Postoperative AF development

The absence of significant difference in relationship between SAT and VAT density and POAF risk between groups (p=0.945 for SAT and p=0.938 for VAT) indicated that these relationships remained consistent regardless of preexisting AF status. Consequently, preoperative AF status was not included as an adjustment factor in the analysis.

In univariable analysis, muscle density (OR 0.929, 95 % CI: 0.871–0.986, p=0.020), VAT density (OR 1.085, 95 % CI: 1.022–1.158, p=0.010), and SAT density (OR 1.071, 95 % CI: 1.012–1.139, p=0.022) showed significant associations with POAF risk.

In the overall cohort, after adjusting for age and gender, both VAT and SAT density emerged as significant predictors of POAF (VAT: OR 1.075, 95 % CI: 1.010;1.149, p=0.026; SAT: OR 1.073, 95 % CI: 1.011;1.146, p=0.025). Analysis of left atrial (LA) dimensions, a traditional risk factor, revealed no significant differences in LA diameter between patients who developed POAF and those who did not (p=0.994), suggesting that tissue quality parameters provided independent prognostic information. Additional adjustment for comorbidities (hypertension, diabetes, respiratory disease) did not meaningfully change these estimates.

Quartile analysis demonstrated a striking gradient of POAF risk across VAT density ranges. Compared to patients in the lowest VAT density quartile (<−106.52 HU), who experienced a POAF rate of 7.7 %, those in the highest quartile (>−99.06 HU) showed a 5.5-fold increased risk with a POAF rate of 42.3 %. This progressive relationship was evident across all quartiles, with Q2 (−106.52 to −102.54 HU) and Q3 (−102.54 to −99.06 HU) showing 5.0-fold and 4.0-fold increased risks respectively, establishing a clear dose-dependent pattern.

SAT density demonstrated a different pattern, with a less consistent gradient. While patients in the lowest SAT density quartile (<−111.82 HU) had a POAF rate of 19.2 %, both Q2 (−111.82 to −107.62 HU) and Q4 (>−104.20 HU) showed equal 2.0-fold increased risks with POAF rates of 38.5 %. Q3 (−107.62 to −104.20 HU) demonstrated only a modest 1.2-fold increased risk with a POAF rate of 23.1 %. These detailed relationships between adipose tissue density parameters and arrhythmia risk are presented in Table S2.

Notably, the analysis revealed statistically significant differences in the relationship between IMAT density and POAF risk between groups (interaction p=0.029). In patients without prior AF (Group A), higher IMAT density showed a positive association with POAF risk (OR 1.167, 95 % CI: 1.011;1.377, p=0.047). In patients with preexisting AF undergoing Maze procedure (Group B), the relationship between IMAT density and POAF development was not statistically significant (p=0.175). Given the modest sample sizes in subgroup analyses, this finding should be considered exploratory and requires validation in larger cohorts.

Detailed subgroup analysis of LA dimensions showed consistent findings across surgical groups. In patients without prior AF history (Group A), median LA diameter was 48 mm (IQR: 43–52 mm) in both those who developed POAF (n=19) and those who did not (n=37). Similarly, in patients with preexisting AF undergoing MAZE procedure (Group B), median LA diameter was 47 mm (IQR: 43–51 mm) in both POAF (n=12) and non-POAF (n=36) subgroups. The nearly identical distribution of LA diameters between groups (p=0.994) strongly suggests that LA size was not a determinant of POAF in our cohort.

Postoperative echocardiographic assessment revealed successful valve repair with minimal residual mitral regurgitation (MR) across the cohort. Due to limited variability in postoperative MR grades (97 % of patients in the same category), statistical evaluation of the association between MR and POAF was not feasible.

### Secondary outcomes and metabolic parameters

Univariable analysis revealed significant associations between tissue quality parameters and perioperative outcomes. Muscle characteristics showed distinct relationships with recovery metrics: higher muscle density correlated with shorter intubation duration (β= −0.072, 95 % CI: −0.121; −0.023, p=0.005), while higher skeletal muscle index predicted shorter ICU stays (β= −0.675, 95 % CI: −1.338; −0.011, p=0.046).

Intramuscular adipose tissue parameters demonstrated opposing effects, with higher IMAT index associated with both increased blood loss (β=24.874, 95 % CI: 5.592;44.156, p=0.012) and prolonged intubation duration (β=0.136, 95 % CI: 0.025;0.248, p=0.017).

However, after adjustment for age and sex, none of these associations maintained statistical significance (all p>0.05), indicating that demographic factors primarily influence these perioperative outcomes.

Clinical characteristics and computed tomography-derived body composition parameters are presented for two patient groups: those without prior AF (Group A) and those with preexisting AF who underwent concurrent Maze procedure (Group B). Values are expressed as mean ± standard deviation for continuous variables and number (percentage) for categorical variables. P-values were calculated using Student’s *t*-test or Mann-Whitney test for continuous variables and Chi-square test for categorical variables. Statistically significant differences (p<0.05) are highlighted in the p-value column. AF = atrial fibrillation; BMI = body mass index; HU = Hounsfield units; IMAT = intramuscular adipose tissue; ICU = intensive care unit; SAT = subcutaneous adipose tissue; SMI = skeletal muscle index; VAT = visceral adipose tissue.

### Long-term outcomes

**Fig. 2:**
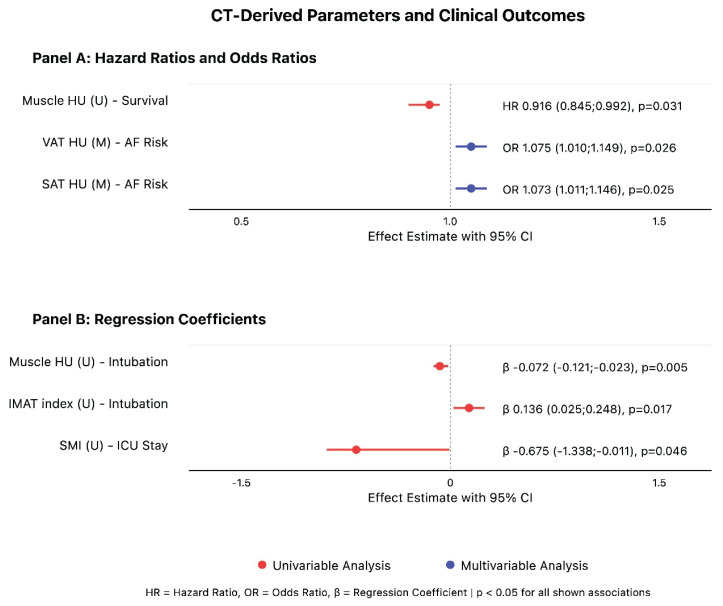
Forest Plot of Association Between CT-Derived Body Composition Parameters and Clinical Outcomes Following Mitral Valve Surgery. Forest plot showing the associations between CT-derived parameters and clinical outcomes. Panel A presents hazard ratios (HR) for overall survival and odds ratios (OR) for AF risk. Panel B presents regression coefficients (β) for secondary outcomes including intubation time and ICU stay duration. Red circles ad lines represent univariable analyses (U), and blue circles and lines represent multivariable analyses (M) adjusted for age and sex. Effect estimates are shown with 95 % confidence intervals (CI). All displayed associations achieved statistical significance (p<0.05). HU = Hounsfield Units; VAT = visceral adipose tissue; SAT = subcutaneous adipose tissue; SMI = skeletal muscle index; IMAT = intramuscular adipose tissue; ICU = intensive care unit.

With median follow-up of 6.17 years (IQR: 3.33–8.38), muscle density emerged as a significant predictor of overall survival in univariable analysis (HR 0.916, 95 % CI: 0.845–0.992, p=0.031). However, in multivariable models adjusted for age and sex, none of the CT-derived tissue parameters maintained independent prognostic significance. Comprehensive analyses of survival outcomes and perioperative complications, including detailed subgroup analyses of tissue-specific parameters, are presented in Tables S3–S5, with key relationships visualized in Figure 2.

## Discussion

Our study highlights three key findings regarding metabolic risk in POAF after minimally invasive mitral valve surgery. First, CT-derived adipose tissue density independently predicts POAF. Second, tissue-quality parameters show distinct associations with POAF depending on prior AF, suggesting different pathophysiological mechanisms. Third, these metabolic markers offer prognostic insights without requiring extra tests beyond routine imaging.

Our analysis underscores the link between tissue-specific metabolic factors and POAF risk in mitral valve surgery [29]. While AF’s multiple re-entry circuits remain foundational, evolving understanding emphasizes the roles of inflammation and metabolic imbalance in both the myocardium and the patient. These processes are particularly relevant after mitral valve procedures, where inflammatory and metabolic pathways interact with arrhythmic substrates.

The strong correlation between adipose tissue density and POAF supports the role of metabolic dysfunction in postoperative arrhythmogenesis [13,14]. Higher VAT and SAT density, indicative of heightened inflammation and metabolic activity [30,31], consistently aligned with increased POAF risk. Notably, patients in the highest VAT density quartile had a 5.5-fold greater POAF risk than those in the lowest quartile, highlighting adipose tissue’s endocrine influence on cardiac electrical stability [32].

While our univariable analyses revealed multiple associations between tissue quality parameters and perioperative outcomes, including relationships between muscle density and intubation duration, IMAT index and blood loss, and reduced SMI with prolonged ICU stays, consistent with previous findings [33,34] most of these relationships were attenuated after adjustment for age and gender. This attenuation suggests that demographic factors significantly influence these perioperative outcomes, highlighting the importance of multivariable analysis in identifying truly independent predictors. This pattern aligns with emerging evidence suggesting that tissue quality parameters, particularly those reflecting adipose tissue metabolism, may provide more reliable perioperative risk indicators than traditional quantitative measures (such as BMI) [35].

The attenuation of tissue parameter associations with survival after multivariable adjustment underscores an important distinction between perioperative and long-term outcomes. While adipose tissue density parameters showed robust independent associations with POAF even after demographic adjustment, their prognostic value for long-term survival appears to be mediated primarily through age-related processes. This pattern suggests that metabolic tissue characteristics may be particularly relevant for acute perioperative complications where metabolic and inflammatory responses play a direct mechanistic role, whereas long-term survival is more heavily influenced by cumulative age-related risk.

Particularly noteworthy is the differential impact of IMAT density on POAF risk between patient groups. The positive association between IMAT density and POAF incidence in AF-naïve patients, but not in those with prior AF, suggests that altered muscle quality may create a distinct metabolically vulnerable substrate for new-onset arrhythmias [36]. However, given the modest sample sizes in this subgroup analysis, this observation should be interpreted cautiously as hypothesis-generating. If validated in larger cohorts, such findings could indicate that the pathophysiological mechanisms underlying first-time POAF may differ from those driving recurrent arrhythmias in patients with pre-existing AF substrates [37].

These relationships reflect complex interactions between cardiometabolic disorders and cardiac electrophysiology. The pathophysiological mechanisms involve adverse atrial remodeling, epicardial adipose tissue deposition, increased inflammatory cytokine production, oxidative stress, and activation of the sympathetic and renin-angiotensin-aldosterone systems [1,38]. Higher adipose tissue density may reflect altered adipokine profiles and inflammatory states that create an arrhythmogenic environment [39].

Our findings complement and extend previous observations from other cardiovascular surgical populations. The prognostic significance of adipose tissue density observed in our mitral valve cohort parallels findings from larger TAVI populations, where Shibata *et al*. (n=1372) demonstrated that higher VAT density correlated with increased mortality risk (HR 1.34, 95 % CI: 1.03;1.76) [39] and Foldyna *et al*. (n=403) reported similar associations for SAT density (HR 1.35, 95 % CI: 1.10;1.67) [10]. While our sample size was smaller, the consistent emergence of tissue quality parameters as significant predictors strengthens their validity as metabolic risk markers.

Our findings also can be contextualized with recent data from other minimally invasive mitral valve surgery cohorts. Notably, in a 2024 multicenter Iberian experience of minimally invasive mitral valve procedures [40] new-onset AF was observed in 15 % of patients during the initial hospital stay, with minimal increase to 17.5 % at one-year follow-up. This relatively low rate of POAF, compared to historical rates of 20–50 % with conventional approaches, suggests that minimally invasive techniques may offer advantages in terms of reducing post-operative arrhythmic complications. However, direct comparisons should be interpreted with caution, as their study did not specify the inclusion or exclusion of concurrent Maze procedures, which could significantly impact the arrhythmia outcomes in their reported population.

## Clinical implications and potential applications

CT-derived adipose tissue density parameters offer several potential advantages for perioperative risk stratification in mitral valve surgery. First, these measurements can be extracted from routine preoperative CT scans already performed for surgical planning, requiring no additional imaging burden or cost beyond automated analysis software. The striking 5.5-fold gradient in POAF risk between the highest and lowest VAT density quartiles suggests that tissue-specific metabolic assessment could substantially enhance current risk prediction models.

Several clinical applications merit consideration. Patients identified as high-risk based on elevated adipose tissue density could be candidates for more intensive perioperative monitoring strategies, including extended continuous telemetry beyond the standard 72-hour period to capture late-onset arrhythmias. Current guidelines support prophylactic antiarrhythmic therapy with beta-blockers or amiodarone in selected high-risk cardiac surgery patients, but patient selection criteria remain imprecise. Integration of CT-derived metabolic parameters with existing clinical risk scores could refine identification of patients most likely to benefit from prophylaxis, potentially improving the risk-benefit balance of these interventions.

From a preoperative optimization perspective, our findings raise intriguing questions about whether metabolic interventions could modify POAF risk in the elective surgical setting. The association between adipose tissue density and arrhythmogenesis suggests that the metabolic quality of adipose tissue, rather than quantity alone, drives risk. Interventions targeting inflammation, insulin resistance, or adipokine dysregulation – through medical therapy, lifestyle modifications, or prehabilitation programs – represent potential strategies for risk reduction, though such approaches would require prospective validation. The differential relationship between IMAT density and POAF in patients with versus without preexisting AF suggests that risk modification strategies may need to be tailored based on underlying arrhythmic substrate.

Implementation barriers include the need for validated automated segmentation tools, establishment of institution-specific reference ranges, and integration into electronic health record systems for real-time risk calculation. Nevertheless, the increasing adoption of artificial intelligence in medical imaging analysis and the routine availability of preoperative CT in cardiac surgery patients create a favorable environment for translation of these findings into clinical practice. Prospective studies are needed to validate these observations, test whether CT-derived metabolic parameters improve risk prediction beyond existing models, and most importantly, determine whether risk-stratified interventions based on these parameters improve clinical outcomes.

## Limitations

Several important limitations warrant consideration. First, the single-center retrospective design may limit generalizability, particularly given the evolution of surgical techniques over the nine-year study period. Second, while our sample size (n=104) was adequate to detect major associations, it limited our ability to perform more granular subgroup analyses and adjust for all potential confounding factors. Additional covariates including hypertension, diabetes mellitus, and respiratory disease were tested but did not materially alter the relationship between CT-derived parameters and POAF risk. The interaction analysis between IMAT density and AF history status, while statistically significant, was based on modest subgroup sizes (n=56 and n=48) that may have limited statistical power to detect true effect modification. This finding should be considered hypothesis-generating and requires validation in larger, independent cohorts before clinical application. The possibility of chance findings due to multiple comparisons in exploratory subgroup analyses cannot be excluded. Third, while left atrial dimensions are traditionally considered a risk factor for AF development, our analysis demonstrated no association between LA diameter and POAF occurrence (p=0.994). This finding suggests that tissue-specific metabolic parameters provide prognostic information independent of conventional anatomical measurements, though validation in larger cohorts is warranted. Our analysis focused specifically on standardized CT-derived metabolic parameters that remain consistent regardless of operator technique. Fourth, our POAF definition (AF lasting >48 h or requiring intervention) with continuous telemetry monitoring for 72 h followed by intermittent ECG may have underestimated the true incidence of postoperative AF, particularly brief paroxysmal episodes occurring after the intensive monitoring period or late-onset AF developing beyond hospital discharge. However, this definition aligns with clinically relevant AF requiring therapeutic intervention and reflects standard practice at our institution. The focus on sustained or clinically significant AF episodes ensures that our findings address arrhythmias with meaningful clinical impact rather than transient, self-limiting episodes. Fifth, precise timing between CT acquisition and surgery was not systematically recorded; however, all scans were performed as part of standardized preoperative protocols without intervening metabolic interventions, and tissue density parameters reflect chronic rather than acute metabolic status. Sixth, Cox proportional hazards modeling revealed violation of proportional hazards assumptions for certain variables (age in univariable analysis, VAT parameters in some multivariable models), which may limit interpretation of hazard ratios, though these violations were marginal. Finally, while CT-derived body composition analysis provides valuable insights, the lack of concurrent biochemical markers limits our ability to fully elucidate the underlying pathophysiological pathways. Future prospective studies should incorporate comprehensive biomarker analyses and standardized imaging protocols to better understand the relationships between metabolic status and arrhythmic risk.

## Conclusions

This investigation reveals that CT-derived adipose tissue density parameters serve as independent predictors of postoperative AF following minimally invasive mitral valve surgery, with 5.5-fold increased risk observed in patients with the highest visceral adipose tissue density. The differential relationship between intramuscular adipose tissue density and arrhythmic risk in patients with and without prior AF provides novel insights into the distinct metabolic substrates underlying primary versus recurrent arrhythmogenesis. By leveraging routine preoperative imaging to assess tissue-specific metabolic status, this approach offers a practical pathway for enhancing perioperative risk stratification without additional testing burden. While these findings establish the potential of CT-derived metabolic parameters in surgical risk assessment, prospective multicenter validation is necessary to define their role in clinical decision-making.

## Supplementary Information





## Figures and Tables

**Table 1:** Comparison of Clinical Characteristics and Body Composition Parameters Between Patients With and Without Preexisting AF (group A and B).

Parameter	Group A: No Prior AF (n=56)	Group B: Prior AF + Maze (n=48)	p-value
*Demographics and Baseline*			

*Age, years*	58.13 ± 11.42	68.66 ± 8.02	< 0.001
*Male sex, n (%)*	38 (67.9)	21 (43.8)	0.023
*BMI, kg/m* *^2^*	27.34 ± 3.91	27.06 ± 3.15	0.683
*LA diameter, mm*	48.0 (43–52)	47.0 (44–51)	0.994

*CT-derived Parameters*			
*Muscle density, HU*	38.60 ± 7.94	34.72 ± 5.97	0.005
*IMAT density, HU*	−67.41 ± 4.55	−70.80 ± 4.72	<0.001
*VAT density, HU*	−101.06 ± 7.13	−100.14 ± 7.12	0.674
*SAT density, HU*	−106.71 ± 7.17	−107.04 ± 8.19	0.708
*SMI, cm* *^2^* */m* *^2^*	50.42 ± 9.33	45.74 ± 7.90	0.007

*Clinical Outcomes*			
*Post-op AF, n (%)*	19 (33.9)	12 (25.0)	-
*Hospital stay, days*	8.86 ± 4.20	8.58 ± 1.89	0.141
*ICU stay, hours*	52.54 ± 30.10	60.51 ± 31.57	0.119
