# Supplementary material for: Post-Operative Atrial Fibrillation: Novel Predictive Value of CT-Derived Adipose Tissue Density in Minimally Invasive Mitral Surgery
Source: Physiol Res. 2025 Dec 1;74(Suppl 1):S117–28. doi: 10.33549/physiolres.935754 (PMC12849796; doi:10.33549/physiolres.935754)
Supplement: Supplementary file 1 [file PR74_S117_Suppl_Fig_1.pdf]

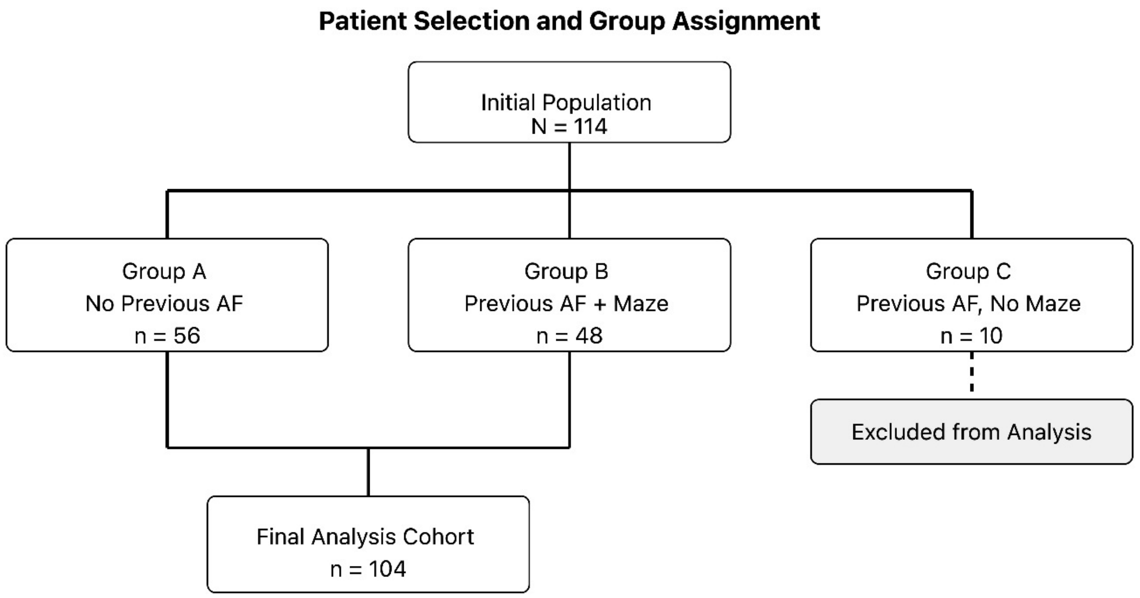

Supplementary Fig. 1. Patient Selection and Stratification Flow Diagram. Patient selection and stratification flow diagram demonstrating the distribution of consecutive patients undergoing minimally invasive mitral valve surgery.
