# Supplementary material for: Post-Operative Atrial Fibrillation: Novel Predictive Value of CT-Derived Adipose Tissue Density in Minimally Invasive Mitral Surgery
Source: Physiol Res. 2025 Dec 1;74(Suppl 1):S117–28. doi: 10.33549/physiolres.935754 (PMC12849796; doi:10.33549/physiolres.935754)
Supplement: Supplementary file 2 [file PR74_S117_Suppl_Tables_1-5.pdf]

Supplementary Table 1. Baseline characteristics and CT-derived body composition parameters in patients undergoing minimally invasive mitral valve surgery.

Characteristic	**Value**			
Demographics			**CT-derived Parameters**	
Age, years	62.99 ± 11.26		**Muscle Parameters**	
Male sex, n (%)	59 (56.7)		- Muscle density, HU	36.81 ± 7.32
Height, cm	171.88 ± 9.87		- SMI, cm^2/m^2	48.26 ± 8.97
Weight, kg	80.45 ± 12.57		**Adipose Tissue Parameters**	
BMI, kg/m^2	27.21 ± 3.57		- IMAT density, HU	-68.97 ± 4.91
Comorbidities			- IMAT index, cm^2/m^2	5.92 ± 3.44
Diabetes mellitus, n (%)	5 (4.8)		- VAT density, HU	-100.63 ± 7.11
Hypertension, n (%)	83 (79.8)		- VAT index, cm^2/m^2	50.97 ± 26.38
Respiratory disease, n (%)	7 (6.7)		- SAT density, HU	-106.86 ± 7.62
Pre-existing AF, n (%)	48 (46.2)		- SAT index, cm^2/m^2	64.07 ± 28.42

Baseline characteristics and computed tomography (CT)-derived body composition parameters are presented for the total study cohort (N=104). CT analysis was performed at the third lumbar vertebra level. Values are expressed as mean ± standard deviation for continuous variables and number (percentage) for categorical variables.

Supplementary Table 2. SAT and VAT density values and post-operative atrial fibrillation occurrence.

VAT (Visceral Adipose Tissue)				
Quartile	**HU Range**	**AF Cases/Total**	**AF Rate**	**Fold vs. Q1**
Q1	<-106.52	2/26	7.70 %	Reference
Q2	-106.52 to -102.54	10/26	38.50 %	5.0× higher
Q3	-102.54 to -99.06	8/26	30.80 %	4.0× higher
Q4	>-99.06	11/26	42.30 %	5.5× higher
SAT (Subcutaneous Adipose Tissue)				
Quartile	**HU Range**	**AF Cases/Total**	**AF Rate**	**Fold vs. Q1**
Q1	<-111.82	5/26	19.20 %	Reference
Q2	-111.82 to -107.62	10/26	38.50 %	2.0× higher
Q3	-107.62 to -104.20	6/26	23.10 %	1.2× higher
Q4	>-104.20	10/26	38.50 %	2.0× higher

Note: This table presents the relationship between adipose tissue density (measured in Hounsfield Units, HU) and post-operative atrial fibrillation (POAF) occurrence. VAT density shows a consistent trend with higher quartiles demonstrating increased POAF risk (up to

5.5-fold in Q4 vs. Q1). SAT density shows a more variable relationship with POAF risk (maximum 2.0-fold increase). Each quartile contains an equal number of patients (n=26), with Q1 representing the lowest density values and Q4 the highest.

Supplementary Table 3. Detailed perioperative outcomes by preoperative AF status.

Outcome	Group A (n=56)		Group B (n=48)	p-value
Mean Intubation Time (hours)	3.86 ± 1.86		4.43 ± 1.89	0.129
Mean ICU Stay (hours)	52.54 ± 30.10		60.51 ± 31.57	0.119
Mean Hospital Stay (days)	8.86 ± 4.20		8.58 ± 1.89	0.141
Blood Loss (ml)	678.21 ± 252.66		902.50 ± 405.31	<0.001

Note: Detailed perioperative outcomes stratified by preoperative atrial fibrillation status. Group A represents patients without previous AF history (n=56); Group B represents patients with preexisting AF who underwent concurrent Maze procedure (n=48). Intubation time is measured from the end of surgery until successful extubation. ICU stay is calculated from admission to discharge from intensive care. Post-operative AF is defined as new onset AF lasting >48 h or requiring intervention. Blood loss volumes were measured intraoperatively. Values are presented as percentages with absolute numbers for categorical variables and mean ± standard deviation for continuous variables. P-values were calculated using Chi-square test for categorical variables and Student's *t*-test or Mann-Whitney test for continuous variables.

Supplementary Table 4. Cox proportional hazards analysis for overall survival.

Variable	Univariable Analysis	p-value	PH Test	Multivariable Analysis†	p-value	PH Test
	HR (95 % CI)		(p-value)	HR (95 % CI)		(p-value)
CT-Derived Muscle Parameters						
Muscle density, HU	0.916 (0.845-0.992)	0.031	0.193	0.986 (0.890-1.093)	0.789	0.169
SMI, cm^2/m^2	0.940 (0.877-1.007)	0.079	0.566	0.927 (0.826-1.040)	0.198	0.140
CT-Derived Adipose Tissue Density						
IMAT density, HU	0.881 (0.770-1.008)	0.066	0.105	0.962 (0.823-1.125)	0.628	0.107
VAT density, HU	1.071 (0.988-1.161)	0.096	0.837	1.081 (0.990-1.180)	0.084	0.043‡
SAT density, HU	1.060 (0.989-1.136)	0.102	0.549	1.076 (0.996-1.161)	0.063	0.124
CT-Derived Adipose Tissue Area Indices						
IMAT index, cm^2/m^2	1.117 (0.976-1.278)	0.107	0.095	0.992 (0.830-1.185)	0.927	0.106
VAT index, cm^2/m^2	0.987 (0.962-1.012)	0.299	0.497	0.981 (0.955-1.009)	0.178	0.028‡
SAT index, cm^2/m^2	1.003 (0.983-1.023)	0.764	0.068	0.990 (0.966-1.014)	0.392	0.106
Demographic and Clinical Variables						
Age, years	1.138 (1.042-1.242)	0.004	0.027‡	-	-	-
Male sex (ref: female)	0.381 (0.117-1.240)	0.109	0.366	-	-	-
BMI, kg/m^2	0.987 (0.836-1.164)	0.872	0.072	-	-	-

Note: Comprehensive Cox proportional hazards analysis for overall survival with median follow-up of 6.17 years (IQR: 3.33-8.38) in 104 patients undergoing minimally invasive mitral valve surgery. The univariable analysis examines each variable independently. The

multivariable analysis adjusts each CT-derived parameter for age and sex simultaneously. Hazard ratios (HR) represent the change in mortality risk associated with one-unit increase in the predictor variable. PH Test refers to the test of proportional hazards assumption (values >0.05 indicate assumptions are met). † Each multivariable model includes one CT-derived parameter plus age and sex as covariates. ‡ Proportional hazards assumption marginally violated (p<0.05); interpret hazard ratios with caution.

Supplementary Table 5. Logistic regression analysis for postoperative atrial fibrillation.

Parameter	**Univariable Analysis**	**p-value**	**Multivariable Analysis†**	**p-value**
	**OR (95 % CI)**		**OR (95 % CI)**	
Combined Cohort Analysis (n=104)				
Muscle density, HU	0.929 (0.871-0.986)	0.020	0.949 (0.875-1.026)	0.196
VAT density, HU	1.085 (1.022-1.158)	0.010	1.075 (1.010-1.149)	0.026
SAT density, HU	1.071 (1.012-1.139)	0.022	1.073 (1.011-1.146)	0.025
SMI, cm^2/m^2	0.974 (0.927-1.022)	0.287	0.992 (0.921-1.067)	0.821
IMAT index, cm^2/m^2	1.094 (0.970-1.237)	0.141	1.023 (0.883-1.184)	0.754
VAT index, cm^2/m^2	0.996 (0.979-1.012)	0.615	0.995 (0.977-1.012)	0.546
SAT index, cm^2/m^2	1.009 (0.994-1.024)	0.239	1.004 (0.986-1.022)	0.661
Age, years	1.046 (1.003-1.096)	0.046	-	-
Male sex (ref: female)	0.618 (0.263-1.441)	0.265	-	-
BMI, kg/m^2	1.089 (0.968-1.231)	0.159	-	-
Subgroup Analysis for IMAT Density‡				
Group A: No prior AF (n=56)				
IMAT density, HU	1.080 (0.954-1.234)	0.236	1.167 (1.011-1.377)	0.047
Group B: Prior AF + Maze (n=48)				
IMAT density, HU	0.825 (0.651-0.986)	0.066	0.860 (0.671-1.046)	0.175
Interaction test (Group × IMAT density)	-	-	-	0.029

Note: Logistic regression analysis for postoperative atrial fibrillation (POAF) in 104 patients undergoing minimally invasive mitral valve surgery. POAF was defined as new-onset atrial fibrillation lasting >48 h or requiring medical intervention during index hospitalization. Odds ratios (OR) represent the change in POAF odds associated with one-unit increase in the predictor variable. † Adjusted for age and sex. Each multivariable model includes one CT-derived parameter plus age and sex as covariates. ‡ IMAT density analyzed separately by group due to significant interaction between IMAT density and preoperative AF status (interaction p=0.029). All other parameters showed no significant interaction (all p>0.05) and were analyzed in the combined cohort.
